# MicroRNA in late lung development and bronchopulmonary dysplasia: the need to demonstrate causality

**DOI:** 10.1186/s40348-016-0047-5

**Published:** 2016-05-23

**Authors:** Claudio Nardiello, Rory E. Morty

**Affiliations:** Department of Lung Development and Remodelling, Max Planck Institute for Heart and Lung Research, Parkstrasse 1, 61231 Bad Nauheim, Germany; Department of Internal Medicine (Pulmonology), University of Giessen and Marburg Lung Center (UGMLC), member of the German Center for Lung Research (DZL), Giessen, Germany

**Keywords:** MicroRNA, miR, Alveolarization, BPD, Bronchopulmonary dysplasia, Lung, Hyperoxia, Preterm, Septation, Development

## Abstract

MicroRNA are emerging as powerful regulators of cell differentiation and tissue and organ development. Several microRNA have been described to play a role in branching morphogenesis, a key step in early lung development. However, considerably less attention has been paid to microRNA as regulators of the process of secondary septation, which drives lung alveolarization during late lung development. Secondary septation is severely perturbed in bronchopulmonary dysplasia (BPD), a common complication of preterm birth characterized by blunted alveolarization. A number of studies to date have reported microRNA microarray screens in animal models of BPD; however, only two studies have attempted to demonstrate causality. Although the expression of miR-150 was altered in experimental BPD, a miR-150^−/−^ knockout mouse did not exhibit appreciable protection in a BPD animal model. Similarly, while the expression of miR-489 in the lung was reduced in clinical and experimental BPD, antagomiR and over-expression approaches could not validate a role for miR-489 in the impaired alveolarization associated with experimental BPD. This mini-review aims to highlight microRNA that have been revealed by multiple microarray studies to be potential causal players in normal and pathological alveolarization. Additionally, the challenges faced in attempting to demonstrate a causal role for microRNA in lung alveolarization are discussed. These include the tremendous variability in the animal models employed, and the limitations and advantages offered by the available tools, including antagomiRs and approaches for the validation of a specific microRNA-mRNA interaction during lung alveolarization.

## Introduction

The development of the mammalian lung is broadly divided into two phases: *early* and *late* lung development. Early lung development occurs primarily in utero and involves the separation of the early respiratory tract from the foregut, and the branching of the conducting airways, along with the associated vasculature [1]. Late lung development is largely concerned with alveolarization: the formation of the alveolar airspaces, which are the principal gas exchange units of the lung. Alveolarization commences with the *saccular* stage of lung development, where the distal airways form saccular units at 24–38 weeks post-conception in humans and embryonic day (E)18 to postnatal day (P)4 in mice. This is followed by the *alveolar* stage of lung development, where the saccular units are subdivided by secondary septa, by the process of secondary septation [2]. This occurs from approximately 32 weeks post-conception in humans, where the bulk of postnatal alveolarization is undertaken within the first 2 years of life; however, some degree of alveolarization may persist into early adulthood [3]. In mice, secondary septation initiates at P4 and is thought to be largely complete by P28; however, some evidence suggests that a slower rate of alveolarization continues for several months [4]. Late lung development is a highly coordinated sequence of events, and any disturbances to the process of alveolarization result in severe perturbations to lung structure. This is exemplified by bronchopulmonary dysplasia (BPD), which is the most common complication of preterm birth and which occurs in infants that receive oxygen supplementation for acute respiratory failure [5]. In affected infants, oxygen toxicity and baro- and volu-trauma associated with mechanical ventilation are believed to disrupt key regulatory pathways that drive alveolarization. This results in a pronounced impairment of late lung development, which generates lungs that have fewer, larger alveoli, along with thickened alveolar septa and a dysmorphic pulmonary vasculature. The process of late lung development is very poorly understood but involves the coordinated action of growth and transcription factors, extracellular matrix (ECM) remodelling, cell differentiation, and physical forces [6–9]. Little is known about the regulation of—and integration of—these processes; however, amongst the emerging candidate regulators of late lung development are microRNA.

## MicroRNA in early lung development

MicroRNA are a relatively new family of small, non-coding RNA that play key roles in animal and plant development, by regulating gene expression [10]. This is also true of the lung, where several studies have already implicated and validated roles for microRNA in early lung development. Early studies revealed that the endoribonuclease Dicer, which processes pre-microRNA to mature microRNA, was required for lung epithelial morphogenesis [11], providing indirect evidence of a role for microRNA in early lung development. Subsequent microRNA microarray screens have identified several microRNA candidates, such as miR-127, as possible players in early lung development [12]. Similar microarray studies have also highlighted sex-specific microRNA expression profiles that are engaged in mice during early lung development and which may explain the impact of sex on aspects of normal and aberrant lung development [13]. Confirmed roles for some microRNA in early lung development have also been established using embryonic lung explants and over-expression and gene deletion studies in mice in vivo. The miR-17-92 (Mirc1) cluster, which includes multiple microRNA, is known to be highly expressed during early lung development, but expression declines as development proceeds. Over-expression of the miR-17-92 cluster in alveolar epithelial cells (under control of the *Sftpc* promoter) led to an abnormal lung and a lethal phenotype and demonstrated that the miR-17-92 cluster promoted a high proliferation rate and an undifferentiated phenotype of lung epithelial progenitor cells [14]. Building on this idea and using mouse lung explants, the miR-17 family was implicated in epithelial bud morphogenesis through modulation of fibroblast growth factor (FGF)-10 signalling [15], which, along with reports (also using mouse lung explants) that miR-140 modulated FGF-9 activity during early lung development [16], highlighted multiple microRNA as regulators of FGF function in the developing lung. As with the miR-17-92 cluster, the miR-302-367 cluster (Mirc20) is also expressed at early stages of lung development, and expression levels decline rapidly as development proceeds. Using gain-of-function and loss-of-function studies in the alveolar epithelium (under the control of the *Sftpc* promoter) in vivo, the miR-302-367 cluster was demonstrated to control multiple aspects of lung endoderm progenitor cell behaviour that directed the formation of a single-layered epithelium, which is ultimately required for functional gas exchange capacity [17]. Staying with the epithelium, several microRNA have also been implicated in foetal alveolar type II cell differentiation, including the miR-200 family [18, 19], miR-375 [20], and miR-124 [21]. The mesenchyme has also received attention, where, using embryonic lung explants, miR-142-3p was implicated in the proliferation and differentiation of mesenchymal cells during early lung development and in Wnt/β-catenin signalling [22]. A role for miR-142 in the modulation of Wnt/β-catenin signalling in the lung was then confirmed in vivo, using miR-142^−/−^ mice [23]. In addition to Wnt/β-catenin signalling, activity of the sonic hedgehog pathway was modulated by miR-326 in embryonic mouse lung explants, which may influence epithelial-mesenchymal cross-talk during early lung development [24]. To date, only one study has addressed a role for microRNA in vascular development during branching morphogenesis of the lung, where miR-221 and miR-130a appear to have opposing effects on tube formation and migration of mouse foetal lung endothelial cells [25]. Thus, microRNA are emerging as master regulators of epithelial, fibroblast, and endothelial contributions to early lung development.

## MicroRNA in late lung development

**Fig. 1:**
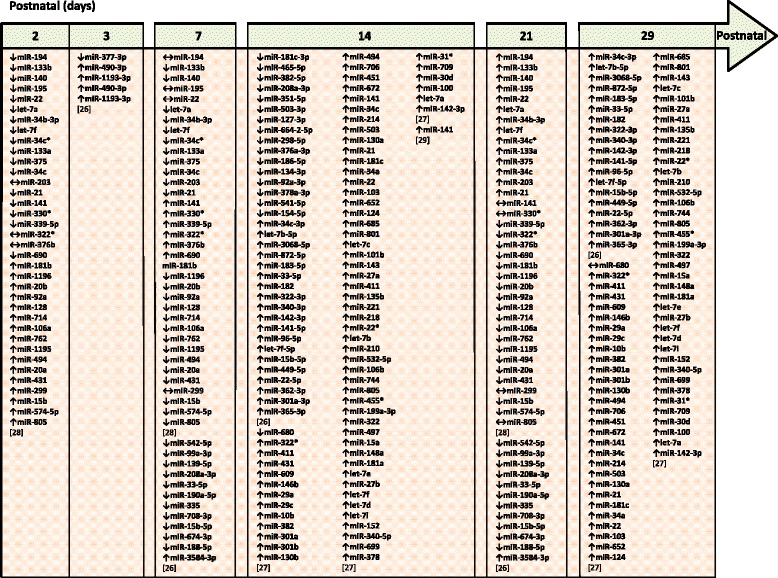
MicroRNA that exhibit altered pulmonary expression identified by microarray studies in experimental bronchopulmonary dysplasia. Changes in microRNA expression are denoted by ↑ (for upregulation), ↓ (for downregulation), or ↔ (no change). MicroRNA nomenclature (for example, miR-34c versus miR-34c* and miR-34b-3p versus miR-34b-5p) have been adopted from the source articles and have not been standardized here. The source articles are described by a number in *square brackets*

**Fig. 2:**
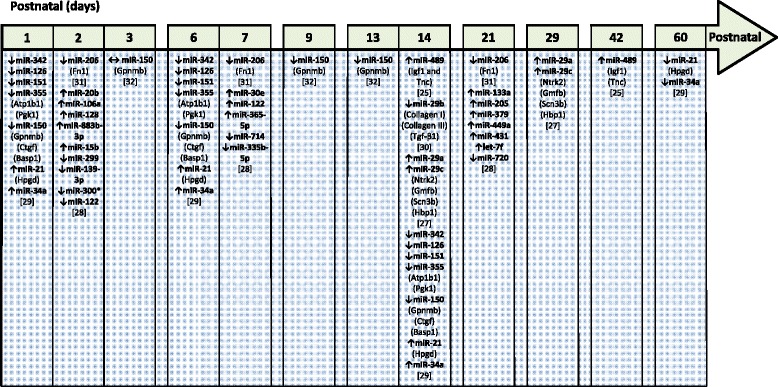
MicroRNA that are proposed a play a role in aberrant lung alveolarization associated with experimental bronchopulmonary dysplasia. The data reported here summarize trends the expression of selected microRNA (in *bold*) that have been noted in microarray studies and that were independently validated (by real-time RT-PCR). Additionally, targets that have been validated by real-time RT-PCTR or immunoblot are also indicated in *parentheses*. Changes in gene expression are denoted by ↑ (for upregulation), ↓ (for downregulation), or ↔ (no change). MicroRNA nomenclature (for example, miR-300 versus miR-300* and miR-139-3p versus miR-139-5p) have been adopted from the source articles and have not been standardized here. The source articles are described by a number in *square brackets*

While a number of detailed descriptive and functional studies have been undertaken during early lung development (outlined above), considerably less work has been undertaken addressing roles for microRNA in alveolarization. Initial hints at candidate microRNA mediators of alveolarization have been obtained using microRNA microarrays to identify temporal changes in the expression of microRNA over the course of normal and aberrant alveolarization, utilizing hyperoxia exposure [26–30], or hyperoxia exposure after sterile (bacterial lipopolysaccharide (LPS)-driven) maternal inflammation [31] in animal models of BPD (“experimental BPD”). MicroRNA exhibiting changes in expression in the lung in response to hyperoxia are listed in Fig. 1. MicroRNA that are deregulated in experimental BPD and that have been independently validated by real-time RT-PCR and/or immunoblot are described in Fig. 2, together with validated microRNA targets. To date, three microRNA have received particular attention in the context of BPD: miR-206 [32], miR-150 [33], and miR-489 [26].

The expression levels of miR-206 were reduced in both experimental BPD in mice and in clinical (human) BPD [32]. Fibronectin-1 (Fn1) was confirmed as a target of miR-206 in lung epithelial cells, and consistent with this idea, Fn1 expression was inversely correlated with miR-206 levels. While these data highlight miR-206 as a candidate mediator of impaired alveolarization associated with BPD, a causal role remains to be established.

A microRNA microarray also identified miR-150 as being deregulated in experimental (hyperoxia-driven) BPD, where lung expression of miR-150 was reduced [30]. Additionally, glycoprotein nonmetastatic melanoma protein b (Gpnmb) was identified and validated as a target of miR-150 [30]. These data suggested that decreased miR-150 levels would lead to increased Gpnmb expression, which was indeed observed in experimental BPD [33]. Consistent with this idea, miR-150^−/−^ mice exhibited increased Gpnmb expression. When miR-150^−/−^ mouse pups were exposed to hyperoxia, a moderate protection of the lung structure was noted at P9; however, no protection was evident at P13, where miR-150^−/−^ mice and wild-type mice exhibited a comparable blunting of alveolarization in response to hyperoxia exposure. Furthermore, the lung capillaries in miR-150^−/−^ mouse pups appeared dysmorphic. These data provide limited support for a causal role for miR-150 in the aberrant lung development associated with experimental BPD.

The expression of miR-489 in the lung was downregulated in both experimental (mouse) and clinical (human) BPD [26]. Both insulin-like growth factor (IGF)-1 and tenascin C (Tnc) were identified as targets of miR-489, and the expression of both IGF-1 and Tnc was increased in the lung in experimental and clinical BPD, ostensibly due to downregulation of miR-489 expression. These data suggested that miR-489 may antagonize secondary septation. However, blocking miR-489 function with a locked nucleic acid antagomiR did not phenocopy the effects of hyperoxia, and the use of antagomiR-489 in vivo in the experimental BPD model paradoxically improved lung structure. Also, contrary to expectations, over-expression of miR-489 from a plasmid delivered to developing mouse lungs worsened lung structure. As such, although miR-489 levels were downregulated in both clinical and experimental BPD, it appeared that miR-489 was a negative regulator of secondary septation. This study made the very important point that change in the expression of microRNA under pathological conditions (for example, BPD) suggests the possibility but does not confirm a de facto role for the affected microRNA as a pathogenic mediator. This study underscored the importance of demonstrating a causal role in lung alveolarization for any microRNA that exhibits expression changes in experimental BPD models.

Clearly, hyperoxia is not the only contributing causal factor in the aberrant lung growth observed in clinical and experimental BPD, where, for example, mechanical forces (generating stretch) due to mechanical ventilation are also an important factor that drives pathology [34, 35]. There is a growing body of evidence that microRNA may also mediate the effects of physical forces on lung development, including normal and pathological alveolarization. For example, in the context of asthma, it has been demonstrated that miR-155 mediated the effects of mechanical stretch on dynamic changes in the secretome of human bronchial epithelial cells [36]. Indeed, an unbiased microarray screen has revealed that cyclic stretch can modulate the expression levels of a broad spectrum of microRNA in vitro, possibly contributing to stretch-associated alveolar epithelial cell dysfunction [37]. However, a connection between microRNA and mechanical forces in lung alveolarization has not yet been documented. Some data have also been generated that addressed microRNA dynamics in developing lungs from rat pups that underwent intrauterine growth restriction, caused by bilateral uterine artery ligation. In these pups, perturbations to the expression levels of miR-132 were noted, and miR-132 was proposed to play a role in disturbing methyl CpG binding protein 2 expression and, hence, impact the epigenetic regulation of alveolarization [38]. Thus, microRNA, in addition to mediating the impact of hyperoxia on lung alveolarization, may also mediate the effects of other injurious stimuli that impact normal lung development.

It is important to consider that hyperoxia exposure or other causal factors that drive aberrant lung alveolarization may concomitantly modulate microRNA expression that, while without a direct functional impact on lung development per se, may participate in pathogenic processes related to comorbidities noted in BPD patients. By way of example, microRNA that are produced in the alveolar epithelium play a critical role in the infection cycle of respiratory syncytial virus (RSV) [39]. Similarly, microRNA have an established role in the development of pulmonary arterial hypertension, which often complicates BPD [40]. As such, injurious stimuli that drive perturbations to microRNA dynamics in the lung and which cause BPD may also facilitate the pathogenesis of microRNA-dependent comorbidities. Conversely, alterations to microRNA expression by co-morbidities may also directly impact lung alveolarization, since, for example, RSV infection itself can alter microRNA expression in human lung cells [41].

Studies on microRNA expression levels in peripheral blood from very low birth weight preterm infants with BPD have identified four microRNA that may serve as biomarkers of BPD [42]. Of these four candidates, two microRNA, namely miR-7 and miR-133b, exhibited increased circulating levels in peripheral blood, while miR-30a-3p and miR-152 levels were reduced. The potential relevance of these four microRNA to the impaired alveolarization associated with BPD has not been established.

In view of these data, no causal role for any microRNA in normal or aberrant alveolarization has yet been confirmed. Many additional candidates exist that have been identified in the microarray studies described in Fig. 2, most notable amongst these is the miR-29 family, which has emerged in multiple studies. The outcome of these causal studies is eagerly awaited.

## Perspective: future work

Despite a large number of potential microRNA mediators of alveolarization, it is clear that a pressing priority is to assign causality and demonstrate a functional role for these microRNA in normal or aberrant lung alveolarization. In the pursuit of this goal, the studies outlined above have highlighted several potential pitfalls in how investigators perform the necessary analyses. These pitfalls are largely related to the models employed. For example, Velten et al. [31] described increased expression of miR-29b in the lung in response to hyperoxia (85 % O_2_ for the first 14 days of life), while, in an alternative model which entailed maternal administration of LPS to mimic chorioamnionitis, prior to exposure of mouse pups to hyperoxia (85 % O_2_ for the first 14 days of life), an opposite trend (i.e. downregulation) of miR-29b expression in the lungs was noted. These data highlight the critical importance of the selection of the BPD model employed.

Along these lines, establishing a consensus about which microRNA are deregulated in experimental BPD models is sometimes problematic due to diverse nature of the experimental BPD models employed. There is diversity in the rodent species (rat [27, 30] versus mouse [26, 28, 29, 31–33]), rodent strain (Kunmin [29, 32], C57BL/6J [26, 33], C3H/HeN [31], ICR [28] mice, and Wistar [27] or Sprague-Dawley [30] rats), and the duration and intensity of oxygen exposure (60 to 100 %, applied either from within 12 h of birth or from P3 or P4; with or without a post-hyperoxia recovery period in normoxia, of varying durations), with or without the pre-application of other injurious stimuli such as LPS [31]. These concerns about the lack of standardization of experimental BPD models have recently been reviewed in detail by Silva et al. [8]. This variability in experimental BPD studies must be kept in mind when attempting to assign a causal role for selected microRNA in alveolarization.

To date, only two studies have examined a causal role for microRNA in experimental BPD, which addressed miR-150 [33] and miR-489 [26]. One study utilized an antagomiR approach, and another employed a global knockout approach. While interesting, it must be kept in mind that antagomiRs have considerable off-target effects and may also neutralize related, relevant microRNA with conserved “seed” sequences, although the antagomiR approach offers an easily applied and stable means of antagonizing microRNA function in a relatively specific fashion. Both the antagomiR and global knockout approaches effectively create a knockout animal. Given the increasingly evident importance of cell-cell cross-talk and the compartmentalization of physiological processes in the developing lung, the use of conditional, cell type-specific driver lines is encouraged, which would permit the assignment of a specific role to a specific microRNA in a specific cell type in the developing lung. It is also important to appreciate that microRNA (and antagomiRs) likely have a multitude of messenger RNA (mRNA) targets in the lung. Validation of a specific mRNA as a target of a specific microRNA provides evidence of a possible causal relationship but does not validate causality. To this end, the introduction of Target Site Blocker (Exiqon) or Target Protector (Qiagen)technology now permits the validation of a specific microRNA-mRNA pair interaction in physiological processes, although these approaches have not yet been applied to the study of lung alveolarization.

Ultimately, the correlation of data generated in experimental animal models must be validated in human studies, if the microRNA in question are to be proposed as a pathogenic mediator of clinical BPD. Along these lines, an exciting recent study by Rogers et al. [43] is the first study in clinical material from BPD patients to demonstrate that the expression levels of all members of the miR-17-92 (Mirc1) cluster were downregulated in autopsied lung material from BPD patients and that the abundance of miR-17 and miR-19b was reduced in the plasma of infants that developed BPD. This is the first study that points to potential causal role members of the miR-17-92 (Mirc1) cluster of microRNA in clinical BPD. These studies will no doubt be strengthened by the increasingly broad spectrum of “-omics” methodologies currently available to the research community [44].

Several recent studies have aimed to improve our analysis of the roles for microRNA in lung development, including a careful analysis of appropriate reference genes for real-time RT-PCR studies [45]. Additionally, efforts have been made to develop computational methods for the integration of microRNA, microRNA targets, and transcription factors into biologically relevant pathways, circuits, and networks [46, 47]. These and other technological advances will no doubt assist us in better understanding the physiological roles of this new and exciting family of non-coding RNA in lung development.
